# Enzymology of the pathway for ATP production by arginine breakdown

**DOI:** 10.1111/febs.15337

**Published:** 2020-05-05

**Authors:** Tjeerd Pols, Shubham Singh, Cecile Deelman‐Driessen, Bauke F. Gaastra, Bert Poolman

**Affiliations:** ^1^ Department of Biochemistry Groningen Biomolecular Sciences and Biotechnology Institute & Zernike Institute for Advanced Materials University of Groningen The Netherlands

**Keywords:** arginine deiminase, arginine deiminase pathway, arginine/ornithine antiporter, carbamate kinase, enzyme kinetics, *Lactococcus lactis*, ornithine transcarbamoylase

## Abstract

In cells, the breakdown of arginine to ornithine and ammonium ion plus carbon dioxide is coupled to the generation of metabolic energy in the form of ATP. The arginine breakdown pathway is minimally composed of arginine deiminase, ornithine transcarbamoylase, carbamate kinase, and an arginine/ornithine antiporter; ammonia and carbon dioxide most likely diffuse passively across the membrane. The genes for the enzymes and transporter have been cloned and expressed, and the proteins have been purified from *Lactococcus lactis* IL1403 and incorporated into lipid vesicles for sustained production of ATP. Here, we study the kinetic parameters and biochemical properties of the individual enzymes and the antiporter, and we determine how the physicochemical conditions, effector composition, and effector concentration affect the enzymes. We report the *K*_M_ and *V*_MAX_ values for catalysis and the native oligomeric state of all proteins, and we measured the effect of pathway intermediates, pH, temperature, freeze–thaw cycles, and salts on the activity of the cytosolic enzymes. We also present data on the protein‐to‐lipid ratio and lipid composition dependence of the antiporter.

## Graphical abstract

In cells, the breakdown of arginine to ornithine and ammonium ion plus carbon dioxide is coupled to the generation of ATP. The proteins involved in arginine breakdown and ATP production include the following: arginine deiminase, ornithine transcarbamoylase, carbamate kinase, and an arginine/ornithine antiporter. We report the kinetic parameters of all the reactions, the native oligomeric state of the proteins, and the effect of pathway intermediates, pH, temperature, freeze–thaw cycles, salts, and lipids on the enzymes.

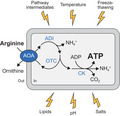

**Abbreviations**
ADI, ArcA: arginine deiminase
AOA, ArcD2: arginine/ornithine antiporter
Carbamoyl‐Pi: carbamoyl‐phosphate
CK, ArcC1: carbamate kinase
DAMO: 2,3‐butanedione monoxime
DOPC: 1,2‐dioleoyl‐*sn*‐glycero‐3‐phosphocholine
DOPE: 1,2‐dioleoyl‐*sn*‐glycero‐3‐phosphoethanolamine
DOPG: 1,2‐dioleoyl‐*sn*‐glycero‐3‐phospho‐(1′‐rac‐glycerol)
KCl: potassium chloride
*K*_eq_: equilibrium constant
KPi: potassium phosphate
*M*_W_: molecular weight
NaCl: sodium chloride
NaPi: sodium phosphate
OTC, ArcB: ornithine transcarbamoylase
PDB: protein database
*pI*: isoelectric point
SEC‐MALS: size‐exclusion chromatography coupled to multi‐angular light scattering

## Introduction

**Fig. 1:**
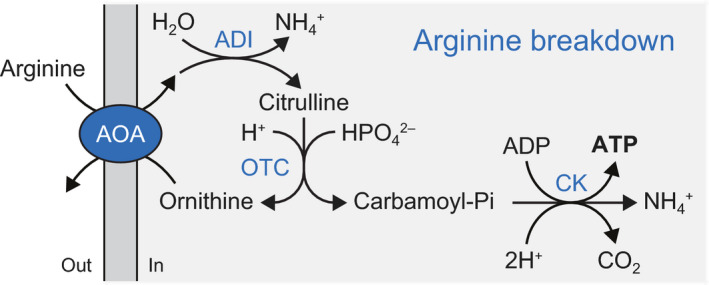
Schematic of the arginine breakdown pathway. AOA, arginine/ornithine antiporter; ADI, arginine deiminase; OTC, ornithine transcarbamoylase; CK, carbamate kinase.

The arginine deiminase pathway is one of the simplest routes for the generation of ATP and alkalinization of the internal pH. With only three cytosolic enzymes, arginine is converted into ornithine, ammonium ion plus carbon dioxide, while ATP is created from ADP and phosphate (Fig. 1); the reaction equation is:

$$\text{L-Arginine}+H_{2}O+{\text{HPO}}_{4}^{2-}+{\text{Mg-ADP}}^{1-}+3H^{+}\to \text{L-Ornithine}+{\text{Mg-ATP}}^{2-}+2{\text{NH}}_{4}^{+}+{\text{CO}}_{2}.$$

The enzymes of the pathway are arginine deiminase (ADI), which hydrolyzes arginine into citrulline plus ammonium ion; ornithine transcarbamoylase (OTC), which converts citrulline plus phosphate into carbamoyl‐phosphate (carbamoyl‐Pi) plus ornithine; and carbamate kinase (CK), which hydrolyzes carbamoyl‐Pi to form carbon dioxide plus ammonium ion under concomitant formation of ATP from ADP and the phosphate moiety of carbamoyl‐Pi. The ADI pathway also employs a membrane‐bound arginine/ornithine antiporter (AOA) to couple the import of the substrate arginine to export of the product ornithine.

The ADI pathway is widely used in bacteria to generate metabolic energy [1, 2] and to protect cells in acidic environments [3, 4]; per molecule of arginine metabolized three protons are used (see reaction equation). The enzymes of the pathway are also found in archaea [5] and lower eukaryotes [6], and some are also present in mammalian cells (based on our own genome searches). In some protozoa, the pathway is used for energy generation [7, 8] but is also important for pathogenesis [9, 10]. By secreting ADI and OTC into the external medium, the concurrent depletion of arginine reduces the production of antiparasitic (and antimicrobial) nitric oxide in infected tissues [9, 10]. Some bacteria have an anabolic OTC in addition to the catabolic one, which is used for arginine biosynthesis instead of arginine breakdown [11]. Interestingly, a catabolic OTC can be changed into an anabolic OTC with only one mutation [11]. The anabolic OTCs have a strongly reduced cooperativity and lower apparent *K*_M_ for carbamoyl‐Pi. In mammalian cells, arginine metabolism is rather complex, as arginine is involved in synthesis of proteins, urea, creatine, polyamines, nitric oxide, proline, glutamate, and agmatine [12]. Mammalian cells use arginases that catalyze the reaction of arginine plus water into ornithine plus urea, and they have anabolic but not catabolic OTCs [13]. Furthermore, mammalian cells do not use CKs but have AOAs in their mitochondria and make use of arginine uniporters [14, 15].

The proteins for breakdown of arginine are encoded by *arcA* (ADI), *arcB* (OTC), *arcC* (CK), and *arcD* (AOA), and in bacteria, they are usually present in a gene cluster [16], although the arrangement of the genes varies between species. In archaea, the proteins have been found in a gene cluster but also dispersed over the chromosome [16]. The *arc* operon in *Lactococcus lactis* contains two copies of *arcC* and *arcD* (due to gene duplication) as well as *arcT*, a putative amino acid transaminase [17], and *argS*, an arginine‐tRNA ligase [18, 19]. Additionally, the transcription factor, *argR*, and an sRNA (*argX*) are located upstream of the *arc* operon, which are arginine‐dependent regulators of the pathway [20, 21]. The *arc* operon is further regulated by the transcription factors CcpA and AhrC, which respond to the concentrations of a preferred sugar (usually glucose) and arginine, respectively [20, 22].

Individual enzymes for arginine breakdown have been studied, but to our knowledge a comprehensive and systematic characterization of all enzymes from a single organism has not been carried out. One study characterizes ADI and CK from *Streptococcus pyogenes* [23], while three separate studies report on ADI, OTC, and CK from *Giardia lamblia* [24, 25, 26]. In addition, ADI and OTC from *Pseudomonas aeruginosa* have been studied [11, 27], as well as OTC and CK from *Enterococcus faecalis* [28, 29, 30] and from *Pyrococcus furiosus* [31, 32]. Other studies focus on individual enzymes [33]. Arginine/ornithine antiport activity was first studied in *L. lactis*, both in membrane vesicles [34] and in whole cells [35]. Later, it was shown in whole cells that *L. lactis* has two AOAs, namely ArcD1 and ArcD2, which have a similar affinity for arginine but very different affinities for ornithine, lysine, histidine, and alanine [36]. The archaeal ArcD antiporter from *Halobacterium salinarum* has been studied in membrane vesicles, which showed a similar *K*_M_ but a lower specific activity for arginine than the protein from *L. lactis* [37].

Recently, we used ArcA, ArcB, ArcC1, and ArcD2 from *L. lactis* to construct an ATP‐regenerating system in synthetic vesicles [38]. The system is fed with external arginine and can maintain constant levels of ATP for up to at least 6 h, even when ATP is consumed. The produced ATP has been used to fuel the gated transport of an osmolyte, which allows the vesicles to respond to osmotic stress. We now characterize ArcA, ArcB, ArcC1, and ArcD2 from *L. lactis* IL1403; we determined the oligomeric state and kinetic parameters of enzymatic conversion or transport of the purified proteins and determined the pH and temperature dependence of the enzymes, and the role of small molecule effectors on their catalytic performance.

## Results

### Choice of the proteins for arginine breakdown

We have studied ArcA, ArcB, ArcC1, and ArcD2 from *L. lactis* IL1403. We chose to work with ArcC1, as initial expression tests showed better production of ArcC1 than ArcC2. We had to work with ArcD2 as ArcD1 from *L. lactis* IL1403 misses the 14th transmembrane helix when aligned to ArcD1 from *L. lactis* MG1363 [39], rendering the transporter inactive (data not shown). The properties of the proteins, including molar extinction coefficient, isoelectric point (*pI*), molecular weights (*M*_W_), and oligomeric state, are summarized in Table 1.

**Table 1:** Properties of the proteins. Values for molar extinction coefficient, isoelectric point (*pI*), and monomeric molecular weight (*M*_W_) were calculated with ExPASy ProtParam. Native *M*_W_ as observed with SEC‐MALS (see Fig. 2B).

Protein	Monomeric molar extinction coefficient (m ^−1^·cm^−1^)	Monomeric *pI*	Monomeric *M*_W_ (kDa)	Native *M*_W_ (kDa)	Oligomeric state
ArcA	34,505	5.45	47.7	194	Tetramer
ArcB	36,900	5.78	40.9	245	Hexamer
ArcC1	21,430	5.08	36.2	73	Dimer
ArcD2	109,445	9.38	56.7	53	Monomer

### Expression and purification of proteins

**Fig. 2:**
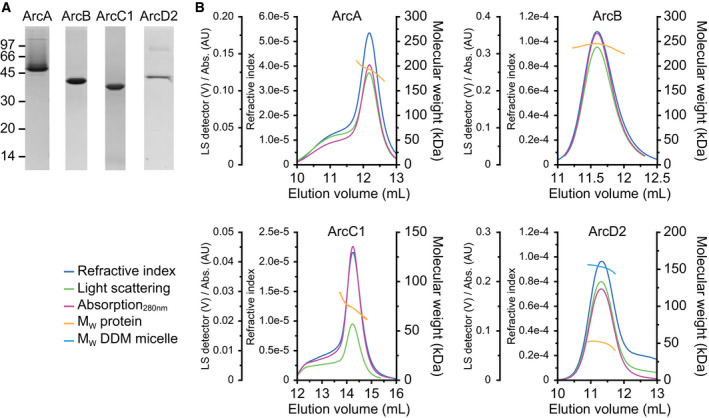
SDS/PAGE and static light scattering analysis of proteins. (A) SDS/PAGE analysis of purified ArcA, ArcB, ArcC1, and ArcD2, of which the theoretical molecular weights of the monomers are 47.7, 40.9, 36.2, and 56.7 kDa, respectively. Full gels are shown in Fig. 7. (B) SEC‐MALS chromatograms of ArcA (top left), ArcB (top right), ArcC1 (bottom left), and ArcD2 (bottom right), showing refractive index (blue traces), light scattering (green traces), and absorption at 280 nm (pink traces). The peaks (yellow traces) indicate molecular weights of 194, 245, 73, and 53 kDa for native ArcA, ArcB, ArcC1, and ArcD2, respectively, which indicates that ArcA is tetrameric, ArcB is hexameric, ArcC1 is dimeric, and ArcD2 is monomeric under native conditions. Additionally, the molecular weight of the DDM mass associated with ArcD2 (cyan trace) is indicated and corresponds to 156 kDa. BSA and aldolase standards are shown in Fig. 8.

ArcA, ArcB, ArcC1, and ArcD2 from *L. lactis* IL1403 were expressed in *L. lactis* NZ9000 (cytosolic proteins) or JP9000 (ArcD2), as described in Ref. [38]. The proteins were purified with metal‐affinity chromatography, followed by size‐exclusion chromatography, which resulted in single bands on a SDS/PAA gel (Fig. 2A). The molecular weights of the SDS‐denatured proteins matched well with the theoretical monomeric molecular weights (ArcA: 47.7 kDa, ArcB: 40.9 kDa, ArcC1: 36.2 kDa, and ArcD2: 56.7 kDa), except for ArcD2, which migrates at a lower molecular weight than expected. This has been observed for many other membrane proteins and is probably due to incomplete denaturation by SDS [40].

### Oligomeric state analysis

We have determined the oligomeric state of the proteins by coupling size‐exclusion chromatography to multi‐angular light scattering (SEC‐MALS), of which the chromatograms are shown in Fig. 2B. SEC‐MALS determines the absolute molar mass and average size of a protein by detecting how it scatters light. For ArcA, we found a native molecular weight of 194 kDa, indicating a tetrameric oligomeric state. The native molecular weight of ArcA from *L. lactis* ATCC 7962 (which has 100% identity with the ArcA studied here) was measured before by Ref. [33]. They found by size‐exclusion chromatography a native molecular weight of 140 kDa, and concluded that the protein is a trimer. We obtained the same molecular weight in our size‐exclusion chromatography analysis (data not shown), but find a higher native molecular weight when the multi‐angular light scattering is included in the analysis. In general, SEC‐MALS is the method of choice to analyze the oligomeric states of proteins, as it allows correcting for differences in shape and, for example, the presence of bound detergent in case of membrane proteins [41]. A structural study on arginine deiminase from *S. pyogenes* (44.5% amino acid identity with *L. lactis* ArcA) showed a mix of monomeric and dimeric states [42], while the protein from *P. aeruginosa* (32.1% identity) showed a tetrameric oligomeric state [43].

The native molecular weight of ArcB was 245 kDa, indicating a hexameric state. Ornithine transcarbamoylase from *Lactobacillus hilgardii* (69.8% identity with *L. lactis* ArcB) and *Gloeobacter violaceus* (35.9% identity) was also found to be hexamers [44]. For ArC1, we found a native molecular weight of 73 kDa, which corresponds to a dimer. Carbamate kinase from *E. faecalis* (48.4% identity with *L. lactis* ArcC1) and *Py. furiosus* (42.6% identity) both showed a dimeric state [32, 45]. Finally, we found a native molecular weight of 53 kDa for ArcD2, which indicates that the arginine/ornithine transporter is monomeric, that is, at least in the DDM‐solubilized state. The arginine/agmatine antiporter AdiC from *Escherichia coli* (22.5% amino acid identity with *L. lactis* ArcD2), which belongs to the same amino acid/polyamine/organocation superfamily of transporters as ArcD2, was found to be a dimer [46].

When analyzing the protein database (PDB) structures of Arc homologs with bound substrates (e.g., ADI from *P. aeruginosa*, 32% identity with *L. lactis* ArcA, PDB ID: 1LXY; OTC from *Vibrio vulnificus*, 48% identity with *L. lactis* ArcB, PDB ID: 4H31; CK from *E. faecalis*, 52% identity with *L. lactis* ArcC1, 2WE5), we find that the proteins have one active site per monomer [45, 47]. Therefore, the *k*_cat_ values were calculated with the assumption that every monomer is catalytically active.

### Characterization of arginine deiminase

**Fig. 3:**
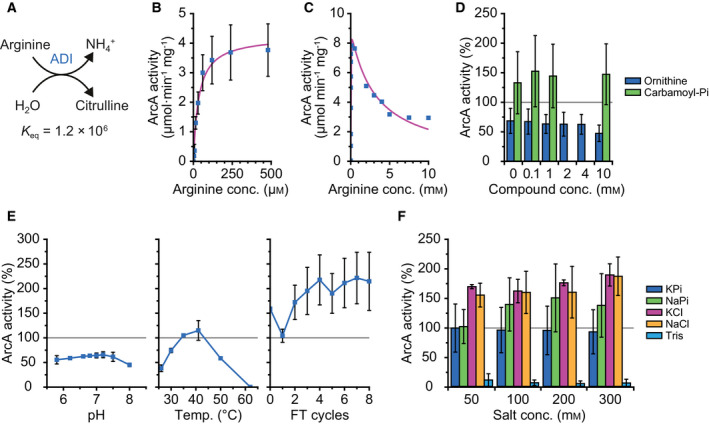
Arginine deiminase (ADI, ArcA) activity. Standard conditions are as follows: 42 nm ArcA, 480 µm arginine in 50 mm KPi, pH 7.0, at 30 °C. Error bars represent the standard deviation between replicates, gray lines mark the activity of ArcA under standard conditions, which is normalized to 100%. (A) Schematic of the ArcA reaction. *K*_eq_ value was calculated with an ionic strength of 0.1 m at pH 7.0 using eQuilibrator 2.2. (B) Michaelis–Menten plot of ArcA activity versus arginine concentration, giving a *K*_M_ of 34.5 ± 6.6 µm, a *V*_MAX_ of 4.2 ± 0.2 µmol·min^−1^·mg^−1^, and a *k*_cat_ of 3.3 ± 0.2 s^−1^. Data from biological triplicates (*n* = 3). (C) Substrate inhibition observed for ArcA at higher arginine concentrations, using 3.2 nm ArcA. Fitted with uncompetitive inhibition (see Materials and methods) gives a *K*_M_ of 5.8 ± 0.9 µm, a *V*_MAX_ of 9.0 ± 0.4 µmol·min^−1^·mg^−1^, and a *K*_I_ of 3.2 ± 0.4 mm. Data from a single experiment (*n* = 1). (D) Influence on ArcA activity of increasing amounts of ornithine (blue bars) and carbamoyl‐Pi (green bars); data from biological duplicates (*n* = 2), 100% activity equals 4.5 µmol·min^−1^·mg^−1^. (E) Influence on ArcA activity of pH (left), temperature (middle), and freeze–thaw (FT) cycles (right); data from biological duplicates (*n* = 2), 100% activity equals 4.2 µmol·min^−1^·mg^−1^. (F) Influence on ArcA activity of different (concentrations of) salts, namely potassium phosphate (KPi; blue bars), sodium phosphate (NaPi; green bars), potassium chloride (KCl; pink bars), sodium chloride (NaCl; yellow bars), and Tris/HCl (Tris; light blue bars). 50 mm KPi was added to all the KCl and NaCl measurements; data from biological duplicates (*n* = 2), 100% activity equals 5.0 µmol·min^−1^·mg^−1^.

We first determined the kinetic parameters of *L. lactis* IL1403 ArcA, using arginine as the substrate (Fig. 3A). The Michaelis–Menten fit of the datapoints is shown in Fig. 3B, giving a *K*_M_ value of 34.5 ± 6.6 µm and a *V*_MAX_ of 4.2 ± 0.2 µmol·min^−1^·mg^−1^ at 30 °C in 50 mm KPi, pH 7.0. The *k*_cat_ was calculated at 3.3 ± 0.2 s^−1^, with the assumption of one active site per monomer, hence four sites per tetramer. When the concentration of arginine is increased beyond 0.5 mm, substrate inhibition is observed with a *K*_I_ of 3.2 mm (Fig. 3C). An earlier study on ArcA from *L. lactis* ATCC 7962 (100% amino acid identity with *L. lactis* IL1403 ArcA) reported a *K*_M_ of 8.7 ± 0.05 mm and a *V*_MAX_ of 345 ± 2 µmol·min^−1^·mg^−1^ at 60 °C in 100 mm KPi, pH 7.2 [33]. These *V*_MAX_ and *K*_M_ values are approximately two orders of magnitude higher than the ones we found, but also much higher when compared to ArcA from other organisms in the BRENDA database (www.brenda‐enzymes.org). We can partly explain the higher *V*_MAX_ and *K*_M_ by the higher temperature at which the ATCC 7962 enzyme was assayed (60 °C *versus* 30 °C), but intriguingly the ATCC 7962 and IL1403 ArcA proteins also differ in temperature dependence (see below).

Kim *et al*. measured citrulline production like we did via the reaction with 2,3‐butanedione monoxime (DAMO), but they did not use a reducing agent (thiosemicarbazide in our assay) or an iron catalyst (we use ammonium iron(III) sulfate) [33, 48]. The ratio between reaction and acid mixture is also different, where we add 150 µL of a 3 : 2 : 5 mixture (v/v) of 95% H_2_SO_4_ and 85% H_3_PO_4_ and H_2_O to 50 µL of reaction mix, and Kim *et al*. [33] add 250 µL of a 1 : 3 mixture (v/v) of 95% H_2_SO_4_ and 85% H_3_PO_4_ to 500 µL of reaction mix. Finally, the concentration of DAMO is very different, as we use about 0.16% (w/v), while the previous study uses 0.75%. We therefore think that their assay is less sensitive to citrulline production than the assay that we used. This could explain why they used 10 mm arginine as the lowest concentration, even though it is higher than the *K*_M_ of 8.7 mm that they report for arginine.

Next, we investigated the effect of ADI pathway intermediates on the activity of ArcA. The addition of up to 10 mm carbamoyl‐Pi did not influence the activity of ArcA (Fig. 3D, green bars), while 10 mm ornithine (20 times in excess of arginine) decreased the activity by 30% (Fig. 3D, blue bars). The inhibition by ornithine is most likely competitive in nature as the amino acid may fit in the same binding pocket as arginine. Carbamoyl‐Pi, on the other hand, is structurally very different and will most likely not compete with arginine for binding to ArcA. Mg‐ATP, Mg‐ADP, Mg‐AMP, or ammonia at a concentration of 2 mm did not affect the activity of ArcA, but 20 mm of ammonia decreased activity by 30% (data not shown).

The optimum pH of ArcA is pH 7.2 at 30 °C (Fig. 3E, left), while the optimum temperature is 41 °C at pH 7.0 (Fig. 3E, middle). Between pH 6.8 and 7.2, the change in activity is small, but lowering the pH to 5.8 decreases activity by 14%, while increasing the pH to 8 drops activity by 30%. When the temperature of the reaction mix was increased from 26 °C to 41 °C, the activity of ArcA increased. Any further increase in temperature led to a decrease in activity, and full inactivation was seen at 62 °C. The same optimal pH of 7.2 was found at 60 °C with ArcA from *L. lactis* ATCC 7962, but in the study of Kim *et al*. [33], the enzyme was completely inactive at pH 6.4 or 7.8. Additionally, Kim *et al*. [33] found an optimal temperature of 60 °C and a decrease of 85% in activity at 40 °C, which is almost the opposite effect of what we observe here. They observed inactivation of the enzyme at 60 °C in about 40 min, while we see that ArcA is completely inactivated within 3 min (Fig. 3E, middle). We have no explanation for the discrepancy between the studies of Kim *et al*. and ours [33].

Freezing the enzyme in liquid nitrogen and subsequently thawing it has little or no effect on ArcA activity (Fig. 3E, right). This finding is important for studies where the enzyme has to be stored or encapsulated into synthetic vesicles, as described by Ref. [38]. Finally, increasing the concentration of potassium phosphate (KPi) does not have an effect on ArcA activity (Fig. 3F, blue bars). Changing to sodium phosphate (NaPi) and increasing its concentration to 100 mm or higher seem to increase ArcA activity, although the error of the measurements was relatively large (Fig. 3F, green bars). Adding 50 mm of potassium chloride (KCl) or sodium chloride (NaCl) to 50 mm KPi had some effect (Fig. 3F, pink and yellow bars), but overall ArcA is not very sensitive to changes in ionic strength. Changing from a phosphate buffer to Tris/HCl reduced activity by 90% (Fig. 3F, light blue bars).

### Characterization of ornithine transcarbamoylase

**Fig. 4:**
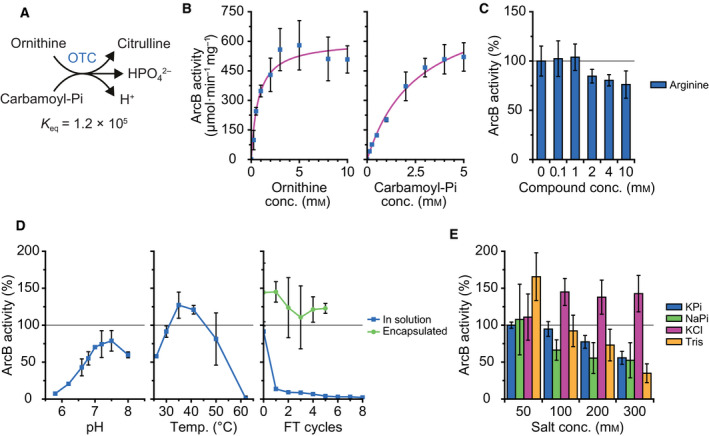
Ornithine transcarbamoylase (OTC, ArcB) activity. Standard conditions are as follows: 1 nm ArcB, 5 mm ornithine, and 5 mm carbamoyl‐Pi in 50 mm KPi, pH 7.0, at 30 °C. Error bars represent the standard deviation between replicates, and gray lines mark the activity of ArcB under standard conditions, which is normalized to 100%. (A) Schematic of the ornithine transcarbamoylase reaction in the direction of citrulline formation, which is opposite of how the reaction runs in the arginine breakdown pathway. *K*_eq_ value was calculated with an ionic strength of 0.1 m at pH 7.0 using eQuilibrator 2.2. (B) The Michaelis–Menten plot of ArcB activity versus ornithine (left) and carbamoyl‐Pi (right) concentration. Fitting gives a *K*_M_ of 0.7 ± 0.2 mm, a *V*_MAX_ of 600 ± 40 µmol·min^−1^·mg^−1^, and a *k*_cat_ of 410 ± 30 s^−1^ when ornithine is varied and carbamoyl‐Pi is kept at 5 mm. Additionally, a *K*_M_ of 2.7 ± 0.4 mm, a *V*_MAX_ of 830 ± 65 µmol·min^−1^·mg^−1^, and a *k*_cat_ of 565 ± 45 s^−1^ are obtained when carbamoyl‐Pi is varied and ornithine is kept at 5 mm; data from biological duplicates (*n* = 2). (C) Influence on ArcB activity of increasing amounts of arginine; data from biological duplicates (*n* = 2), 100% activity equals 340 µmol·min^−1^·mg^−1^. (D) Influence on ArcB activity of pH (left), temperature (middle), and freeze–thaw (FT) cycles (right). Effect of FT cycles was studied for ArcB in solution (blue squares) and encapsulated in proteoliposomes (green circles); data from biological duplicates (*n* = 2), 100% activity equals 480 µmol·min^−1^·mg^−1^. (E) Influence on ArcB activity of different (concentrations of) salts. 10 mm KPi was added to all the KCl measurements; data from biological duplicates (*n* = 2), 100% activity equals 310 µmol·min^−1^·mg^−1^.

The kinetic parameters of *L. lactis* IL1403 ArcB were characterized with ornithine and carbamoyl‐Pi as substrates (Fig. 4A). In the breakdown of arginine, this reaction runs in the opposite direction, but the equilibrium constant (*K*_eq_) favors formation of citrulline plus inorganic phosphate by a factor of ~ 10^5^. The forward reaction has been studied for ArcB from *E. coli*, using arsenate instead of phosphate, yielding the formation of ornithine plus carbamoyl‐arsenate [49]; the latter decomposes spontaneously into carbon dioxide, ammonium ion, and arsenate. The Michaelis–Menten fits of the datapoints of Fig. 4B yield a *K*_M_ value of 0.7 ± 0.2 mm and a *V*_MAX_ of 600 ± 40 µmol·min^−1^·mg^−1^ for ornithine (left panel), when the concentration of carbamoyl‐Pi is kept at 5 mm. When the concentration of carbamoyl‐Pi is varied, we obtain a *K*_M_ of 2.7 ± 0.4 mm and a *V*_MAX_ of 830 ± 65 µmol·min^−1^·mg^−1^ (right panel), with the concentration of ornithine fixed at 5 mm. The *k*_cat_ values are 410 ± 30 s^−1^ and 565 ± 45 s^−1^ for ornithine and carbamoyl‐Pi, respectively, assuming one active site per monomer. When we compare our values with those of other ArcB‐like enzymes in the BRENDA database, we find that the *K*_M_ for carbamoyl‐Pi is the highest, while the *K*_M_ for ornithine is one of the lowest; our *k*_cat_ values are among the highest reported so far.

With 10 mm of arginine (two times more than the concentrations of ornithine and carbamoyl‐Pi), the activity of ArcB decreased by 25% (Fig. 4C). Like the inhibition of ArcA by ornithine, ArcB may be inhibited by arginine because it is structurally similar to ornithine. The addition of 2 mm Mg‐ATP, Mg‐ADP, Mg‐AMP, or 20 mm ammonia had no effect on ArcB (data not shown). The optimum pH of ArcB was pH 7.5 at 30 °C (Fig. 4D, left), and the optimum temperature was 35 °C at pH 7.0 (Fig. 4D, middle). Lowering of the pH from 7.0 to 5.8 inactivated the enzyme for more than 90%, while increasing the pH to 8.0 reduced the activity by 25%. Lowering the temperature from 35 to 25 °C lowered activity by 75%, while increasing the temperature to 62 °C inactivated ArcB, similar to what we observed for ArcA.

We find that ArcB in solution is sensitive to cycles of freezing and thawing (FT) (Fig. 4D, right). After just one FT cycle, we observed an 80% loss in activity. To check whether this inactivation also happens when ArcB is encapsulated in vesicles (as done in Ref. [38]), we mimicked those conditions by adding ArcD2 proteoliposomes, Mg‐ADP, and ornithine to the FT mix; we omitted ArcA and ArcC1 to be able to measure ArcB activity. After a varying number of cycles of freezing and thawing, the vesicles were dissolved with 0.1% (v/v) Triton X‐100 and ArcB activity was determined. Importantly, ArcB in the vesicles was much less affected by freezing and thawing, as a loss of activity of only 25% was observed after five FT cycles. Most likely, the encapsulation of ArcB in the vesicles and the presence of the substrate (ornithine) stabilize the enzyme.

Increasing the concentration of KPi or NaPi decreased the activity of ArcB, probably because the reactant inorganic phosphate becomes inhibitory (Fig. 4E, blue and green bars). This notion is supported by the finding that increasing the salt concentration (addition of KCl) did not inhibit the enzyme. In fact, concentrations of KCl above 50 mm increased the activity of ArcB (Fig. 4E, pink bars). ArcB was inhibited by Tris/HCl at concentrations higher than 50 mm (Fig. 4E, yellow bars).

### Characterization of carbamate kinase

**Fig. 5:**
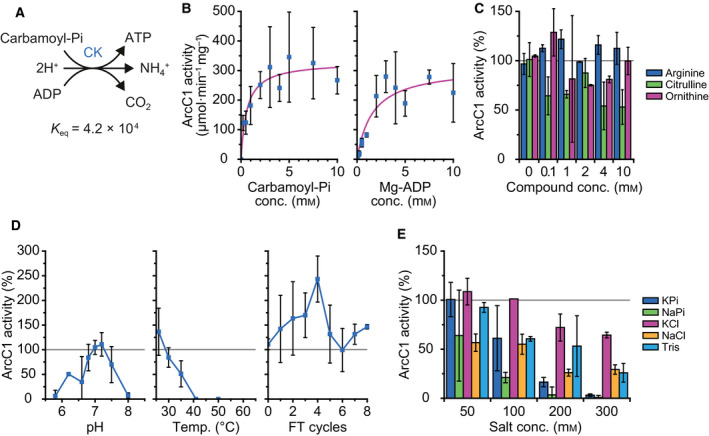
Carbamate kinase (CK, ArcC1) activity. Standard conditions are as follows: 46 nm ArcC1, 5 mm carbamoyl‐Pi, and 5 mm Mg‐ADP in 50 mm KPi, pH 7.0, at 30 °C. Error bars represent the standard deviation between replicates, and gray lines mark the activity of ArcC1 under standard conditions, which is normalized to 100%. (A) Schematic of the carbamate kinase reaction. *K*_eq_ value was calculated with an ionic strength of 0.1 m at pH 7.0 using eQuilibrator 2.2. (B) The Michaelis–Menten plot of ArcC1 activity versus carbamoyl‐Pi (left) and ADP (right) concentration. Fitting gives a *K*_M_ of 0.6 ± 0.2 mm, a *V*_MAX_ of 330 ± 30 µmol·min^−1^·mg^−1^, and a *k*_cat_ of 200 ± 20 s^−1^ when carbamoyl‐Pi is varied and Mg‐ADP is kept at 5 mm. A *K*_M_ of 1.6 ± 0.7 mm, a *V*_MAX_ of 310 ± 40 µmol·min^−1^·mg^−1^, and a *k*_cat_ of 190 ± 15 s^−1^ are obtained when Mg‐ADP is varied and carbamoyl‐Pi is kept at 5 mm; data from biological duplicates (*n* = 2). (C) Influence on ArcC1 activity of increasing amounts of arginine (blue bars), citrulline (green bars), and ornithine (pink bars); data from biological duplicates (*n* = 2); 100% activity equals 390 µmol·min^−1^·mg^−1^. (D) Influence on ArcC1 activity of pH (left), temperature (middle), and freeze–thaw (FT) cycles (right); data from biological duplicates (*n* = 2), 100% activity equals 380 µmol·min^−1^·mg^−1^. (E) Influence on ArcC1 activity of different (concentrations of) salts and buffers. 50 mm KPi was added to all the KCl and NaCl measurements; data from biological duplicates (*n* = 2), 100% activity equals 400 µmol·min^−1^·mg^−1^.

We used carbamoyl‐Pi and Mg‐ADP as substrates to determine the kinetic parameters of the reaction catalyzed by ArcC1 from *L. lactis* IL1403 (Fig. 5A). The Michaelis–Menten fits of the datapoints of Fig. 5B give a *K*_M_ value of 0.6 ± 0.2 mm and a *V*_MAX_ of 330 ± 30 µmol·min^−1^·mg^−1^ for carbamoyl‐Pi (left panel), when the concentration of Mg‐ADP is fixed at 5 mm. For Mg‐ADP, a *K*_M_ of 1.6 ± 0.7 mm and a *V*_MAX_ of 310 ± 40 µmol·min^−1^·mg^−1^ were determined (right panel), when carbamoyl‐Pi is kept constant at 5 mm. The *k*_cat_ values of ArcC1 are 200 ± 20 s^−1^ and 190 ± 15 s^−1^ when carbamoyl‐Pi and Mg‐ADP are varied, respectively, assuming one active site per monomer. The error bars in Fig. 5B are relatively large, as the data were obtained from the combination of two independent ratiometric fluorescent measurements, using PercevalHR for the determination of the ATP/ADP ratio and pyranine for pH. Because the PercevalHR readout is sensitive to pH [38, 50], we used the pyranine data to correct the Perceval HR fluorescence for the increase in pH. According to the BRENDA database, very few ArcC‐type enzymes have been studied. The *k*_cat_ values reported here for ArcC1 are the highest reported up to now.

Adding up to 10 mm of arginine did not negatively affect the activity of ArcC1 (Fig. 5C, blue bars). Citrulline at 10 mm decreased the activity by 40–50% (Fig. 5C, green bars), while ornithine was slightly inhibitory (Fig. 5C, pink bars). The optimum pH for ArcC1 activity is 7.0 at 30 °C (Fig. 5D, left), while the optimum temperature is 25 °C at pH 7.0 (Fig. 5D, middle). The enzyme has a narrow pH window for its activity, as ArcC1 is fully inactivated below pH 5.8 or above pH 8.0. ArcC1 is also much more sensitive to temperature than ArcA and ArcB. Freeze–thaw cycles, on the other hand, had little influence on the activity of ArcC1 (Fig. 5D, right).

KPi and NaPi decreased the activity of ArcC1 when their concentrations were above 50 mm (Fig. 5E, blue and green bars). The inhibition by increasing NaPi or KPi concentration may reflect competitive inhibition as inorganic phosphate may compete with carbamoyl‐Pi for binding to ArcC1. Interestingly, NaPi inhibits the activity of ArcC1 more than KPi does, an effect that is also seen when adding NaCl (70% inhibition at 300 mm) instead of KCl (35% inhibition at 300 mm; Fig. 5E, pink and yellow bars). As observed for ArcA and ArcB, Tris/HCl inhibited ArcC1 at concentrations above 50 mm (Fig. 5E, light blue bars).

### Characterization of the arginine/ornithine antiporter

**Fig. 6:**
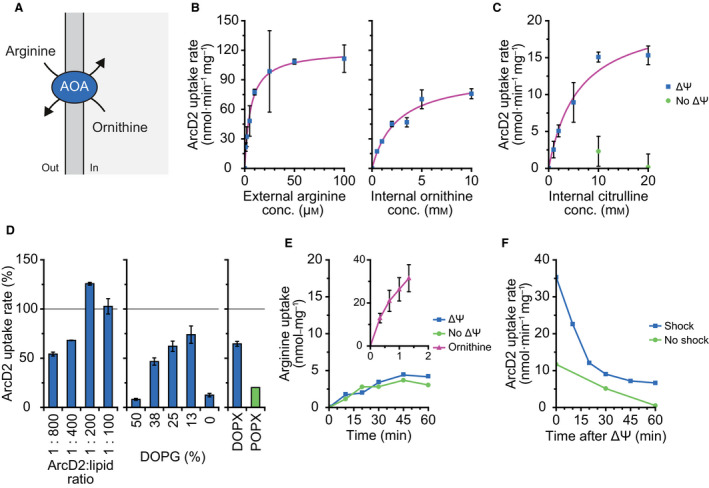
Arginine/ornithine antiporter (AOA, ArcD2) activity. Standard conditions are as follows: 1 : 200 (w/w) ArcD2 : lipid ratio, using 50 : 38 : 12 (w/w) DOPE : DOPG : DOPC lipids extruded through 200 nm polycarbonate filters; in transport assays: 176 nm ArcD2 plus 25 µm arginine externally and 10 mm ornithine or citrulline internally in 50 mm KPi, pH 7.0, at 30 °C. Error bars represent the standard deviation between replicates; gray lines mark the activity of ArcD2 under standard conditions, which is normalized to 100%. (A) Schematic of the arginine/ornithine antiport reaction. (B) The Michaelis–Menten plot of ArcD2 activity versus external arginine (left) and internal ornithine (right) concentration. Fitting gives a *K*_M_ of 6.0 ± 0.7 µm, a *V*_MAX_ of 120 ± 5 nmol·min^−1^·mg^−1^, and a *k*_cat_ of 0.11 ± 0.005 s^−1^ when external arginine is varied and internal ornithine is kept at 10 mm. Additionally, a *K*_M_ of 2.5 ± 0.7 mm, a *V*_MAX_ of 95 ± 10 nmol·min^−1^·mg^−1^, and a *k*_cat_ of 0.09 ± 0.009 s^−1^ are obtained when ornithine is varied and arginine is kept at 25 µm. Arginine data from biological triplicates (*n* = 3), ornithine data from technical duplicates (*n* = 2). (C) The Michaelis–Menten plot of ArcD2 activity versus internal citrulline concentration, with and without a membrane potential (ΔΨ) of −120 mV, giving a *K*_M_ of 6.0 ± 2.1 mm and a *V*_MAX_ of 20 ± 3 nmol·min^−1^·mg^−1^ for measurements in the presence of ΔΨ; data from technical duplicates (*n* = 2). (D) Influence on ArcD2 activity of protein‐to‐lipid ratio (left), lipid headgroups (middle), and lipid tail types (right). Protein‐to‐lipid ratios range from 1 : 800 (w/w), to 1 : 400, 1 : 200, and 1 : 100. Lipid headgroups were varied from 50 : 50 : 0 (w/w) (DOPE : DOPG : DOPC) to 50 : 38 : 12, 50 : 25 : 25, 50 : 13 : 37, and 50 : 0 : 50. The 50 : 38 : 12 lipid composition was studied with DO and PO acyl tails. Data from technical duplicates (*n* = 2), with 10 mm ornithine internally; protein‐to‐lipid ratios data with 25 µm arginine externally; lipid headgroups and tail types data with 10 µm arginine externally; 100% activity equals 120 nmol·min^−1^·mg^−1^. (E) Influx of arginine into empty proteoliposomes, with and without a membrane potential (ΔΨ) of −120 mV. Inset: influx of arginine into proteoliposomes filled with 10 mm ornithine. Empty proteoliposomes data from single experiment (*n* = 1); ornithine data from biological triplicates (*n* = 3), with 1 µm arginine outside. (F) Initiation of the arginine/citrulline antiport reaction after different periods of imposing a membrane potential (ΔΨ) of −120 mV, in the presence (blue squares) and absence (green circles) of an osmotic shock. Data from single experiment (*n* = 1), with 10 mm citrulline internally.

The kinetic parameters of ArcD2 from *L. lactis* IL1403 were determined in proteoliposomes with external arginine as substrate and internal ornithine or citrulline as counter‐substrate for the antiport reaction (Fig. 6A). The standard proteoliposomes consisted of 50 mole% 1,2‐dioleoyl‐*sn*‐glycero‐3‐phosphoethanolamine (DOPE), 38 mole% 1,2‐dioleoyl‐*sn*‐glycero‐3‐phospho‐(1′‐rac‐glycerol) (DOPG), and 12 mole% 1,2‐dioleoyl‐*sn*‐glycero‐3‐phosphocholine (DOPC). The Michaelis–Menten fits of arginine/ornithine antiport are shown in Fig. 6B, giving a *K*_M_ value of 6.0 ± 0.7 µm and a *V*_MAX_ of 120 ± 5 nmol·min^−1^·mg^−1^ for external arginine (left panel) when 10 mm of ornithine is present on the inside. Additionally, a *K*_M_ of 2.5 ± 0.7 mm and a *V*_MAX_ of 95 ± 10 nmol·min^−1^·mg^−1^ were obtained for internal ornithine and 25 µm of arginine on the outside (right panel). The *k*_cat_ values are 0.11 ± 0.005 s^−1^ and 0.09 ± 0.009 s^−1^ when arginine and ornithine, respectively, were varied in the kinetic analysis. Previously, ArcD2 was studied in whole cells [36], who found a *K*_M_ of 4 ± 2 µm and a *V*_MAX_ of 22 ± 17 nmol·min^−1^·mg^−1^ for external arginine. Surprisingly, Noens *et al*. [36] found a 5‐ to 6‐fold higher *V*_MAX_ for ornithine/ornithine than for arginine/ornithine antiport. It is possible that the different lipid compositions, varying internal substrate concentration(s), or the presence of additional arginine and/or ornithine transporters have affected the whole‐cell measurements. We therefore do not further compare our measurements with those in intact cells.

Next, we characterized ArcD2‐mediated arginine/citrulline antiport. Without a membrane potential (ΔΨ), no arginine/citrulline antiport was observed, even with 20 mm internal citrulline (Fig. 6C, green circles). When a ΔΨ of −120 mV was applied (by diluting the KPi‐filled proteoliposomes 100‐fold into NaPi plus valinomycin; see Materials and methods), we observed transport. We determined a *K*_M_ of 6.0 ± 2.1 mm and a *V*_MAX_ of 20 ± 3 nmol·min^−1^·mg^−1^ when internal citrulline was varied with 25 µm arginine on the outside (Fig. 6C, blue squares). For arginine/ornithine antiport, we do not observe an effect of imposing a ΔΨ [38]. The *K*_M_ values for internal ornithine and citrulline are similar, while in the presence of a ΔΨ the *V*_MAX_ for arginine/citrulline antiport is about five times lower than the one found for arginine/ornithine antiport; in the absence of a ΔΨ, the *V*_MAX_ for the arginine/citrulline antiport is below our detection limit. The stimulation of arginine/citrulline antiport by a membrane potential inside negative is in accordance with the difference in charge of arginine (cationic) and citrulline (neutral); hence, this translocation cycle is electrogenic.

We tested the activity of ArcD2 at protein‐to‐lipid ratios (w/w) of 1 : 800, 1 : 400, 1 : 200, and 1 : 100 (Fig. 6D, left). The highest uptake rate was observed at a ratio of 1 : 200, but the highest specific activity was found at 1 : 800, that is, when we assume that equal fractions of ArcD2 are reconstituted at each protein‐to‐lipid ratio. Next, we varied the amount of DOPG (and inversely DOPC) while keeping DOPE constant (Fig. 6D, middle). The extremes of 50 mole% DOPG and 0 mole% DOPG (and thus 0 mole% DOPC and 50 mole% DOPC, respectively) lowered the activity by 80–90%. Lowering the concentration of DOPG from 38 mole% to 25 or 13 mole% however increased the activity by 30% and 60%. Finally, we replaced one of the dioleoyl tails by a palmitoyl tail in the lipid mixture with 38 mole% of PG and 12 mole% PC plus 50 mole% PE. Surprisingly, we find that the activity of ArcD2 in POPX lipids is reduced by 60% when compared to DOPX (Fig. 6D, right). Thus, the increased packing and presumably lower fluidity of membranes composed of POPX lipids reduce the activity of the ArcD2 transporter [51, 52]. We recently made the opposite observation for an eukaryotic amino acid transporter [53].

It has been reported that (small) peptides rich in arginine permeate lipid membranes without the need for a membrane protein [54, 55]. This prompted us to study the passive influx of arginine into vesicles with and without ArcD2 (Fig. 6E) and thus discriminate passive influx from carrier‐mediated uniport. Empty ArcD2 proteoliposomes were incubated with 1 µm of radiolabeled arginine for 1 h, which shows very slow uptake at a rate of about 0.09 nmol·min^−1^·mg^−1^. The same experiment was also performed with liposomes without ArcD2, which showed no arginine uptake at all (data not shown). It thus seems that ArcD2 can uniport arginine slowly, albeit at a rate of no more than 0.4% the speed of arginine/ornithine antiport.

Finally, we determined how long an artificially imposed membrane potential (ΔΨ) lasts in vesicles composed of 38 mole% DOPG and 12 mole% DOPC plus 50 mole% DOPE (Fig. 6F). We diluted ArcD2 proteoliposomes, filled with 10 mm citrulline and 50 mm KPi pH 7.0, into 50 mm NaPi pH 7.0 with valinomycin (see Materials and methods), and after 0, 10, 20, 30, 45, and 60 min, we added radiolabeled arginine. Figure 6C shows arginine/citrulline antiport only when a ΔΨ is present. We find the highest rate of transport when uptake of arginine is assayed from the moment the membrane potential is imposed. As expected, the rate decreases at later times, which reflects the transient nature of the ΔΨ. The membrane potential is maintained for at least 60 min (albeit at a lower value than initially) when the proteoliposomes are osmotically shocked by addition of 250 mm NaCl (Fig. 6F, blue squares). In the unshocked condition, the ΔΨ drops to zero within 60 min (Fig. 6F, green circles).

## Discussion

We report the characterization of the enzymes and transporter responsible for breakdown of arginine and production of ATP from a single organism. As presented in the introduction, this pathway has recently been incorporated into lipid vesicles to form an ATP‐regenerating system that can perform long‐term energy homeostasis [38]. We find that the import of arginine into cells or vesicles by ArcD2 and the concomitant export of the product of arginine breakdown, that is, ornithine, are relatively slow and presumably rate‐determining for the production of ATP. The *k*_cat_ of ArcD2 is more than an order of magnitude lower than that of the slowest enzyme, and the maximal rate of arginine/ornithine transport is reached at a concentration of about 60 µm (*K*_M_ = 6.0 µm). ArcA or ArcB may catalyze the second slowest step. ArcA has a lower *k*_cat_ than ArcB, but the latter enzyme was assayed in the backward reaction; the forward reaction requires a high concentration of citrulline plus inorganic phosphate and a low concentration of ornithine and carbamoyl‐Pi. The high *k*_cat_ of ArcC1 ensures that carbamoyl‐Pi is rapidly converted into ammonia plus carbon dioxide (with concomitant production of ATP from ADP).

One of the first improvements for the ATP‐regenerating system in synthetic vesicles would thus be to incorporate a faster arginine/ornithine antiporter, one with a higher *K*_M_ for arginine and corresponding increase in *k*_cat_. ArcD2 is a well‐coupled antiporter, as arginine uniport occurs at less than 1% of the arginine/ornithine antiport activity. The protein also mediates arginine/citrulline antiport, but this activity is very low in the absence of a membrane potential. These two qualities are important to keep in mind and to test for when looking for alternative arginine/ornithine antiporters.

The enzymes of the pathway are not very sensitive to pathway intermediates, albeit that some inhibition by inorganic phosphate of the backward reaction of ArcB and the forward reaction of ArcC1 is observed. The inhibition of ArcB will however not pose a problem for the breakdown of arginine, because the inorganic phosphate is used as a substrate in the forward reaction of ArcB. The pH and temperature ranges in which the system work optimally are quite narrow, as the activity of the enzymes (in particular of ArcC1) is strongly decreased when deviating from pH 7.0 and 30 °C. A second improvement to the ATP‐regenerating system would be to use a more robust carbamate kinase, that is, an enzyme that is influenced less by pH and temperature.

The freeze–thaw cycles needed for inclusion of the enzymes into vesicles and future synthetic cells do not have significant adverse effects on the reconstitution of the arginine breakdown pathway. This is an important finding, as the freeze–thaw cycles are an essential step in the creation of synthetic cell‐like systems. Furthermore, the enzymes work well in phosphate buffers with potassium or sodium as the counter ion, which are physiological conditions.

The enzymes characterized in this paper have been coupled to ATP consumption by reconstituting the metabolic network with the ATP‐dependent glycine betaine importer OpuA [38]. We have observed that without ATP consumption, the ATP/ADP ratio increases until all ADP is consumed. The full pathway then stops but surprisingly arginine uptake and deamination continues, leading to a futile cycle with acidification of the interior of the cell. With OpuA activity present, the full pathway continues with limited futile hydrolysis of arginine and the ATP/ADP ratio stays constant for several hours. Thus, adding an ATP consuming reaction leads to greater stability (and basic homeostasis) of the pathway, which is what one expects for an out‐of‐equilibrium metabolic network.

## Materials and methods

### Materials

Common chemicals were of analytical grade and ordered from Sigma‐Aldrich Corporation (St. Louis, MO, USA), Carl Roth GmbH & Co., KG (Karlsruhe, Germany) or Merck KGaA (Darmstadt, Germany). The lipids were obtained from Avanti Polar Lipids, Inc. (Alabaster, AL, USA) (> 99% pure, in chloroform): 1,2‐dioleoyl‐*sn*‐glycero‐3‐phosphoethanolamine (DOPE) [850725C], 1,2‐dioleoyl‐*sn*‐glycero‐3‐phosphocholine (DOPC) [850375C], 1,2‐dioleoyl‐*sn*‐glycero‐3‐phospho‐(1′‐rac‐glycerol) (DOPG) [840475C], 1‐palmitoyl‐2‐oleoyl‐*sn*‐glycero‐3‐phosphoethanolamine (POPE) [850757C], 1‐palmitoyl‐2‐oleoyl‐*sn*‐glycero‐3‐phosphocholine (POPC) [850457C], and 1‐palmitoyl‐2‐oleoyl‐*sn*‐glycero‐3‐phospho‐(1′‐rac‐glycerol) (POPG) [840457C]. *n*‐dodecyl‐β‐d‐maltoside (DDM) [D97002] was purchased from Glycon Biochemicals GmbH (Luckenwalde, Germany) and Triton X‐100 [T9284] from Sigma‐Aldrich Corporation. ^14^C‐l‐arginine was purchased from Moravek, Inc. (Brea, CA, USA) [MC‐137, 338 mCi·mmol^−1^].

### Expression and purification of proteins

The construction of strains used for the expression of ArcA, ArcB, ArcC1, and ArcD2 has been described in Ref. [38], together with the methods for expression, purification, and storage of the proteins. For the characterization of ArcD2, reconstitution was done as described for the ^14^C‐l‐arginine transport assays.

### Protein purity and concentration determination

**Fig. 7:**
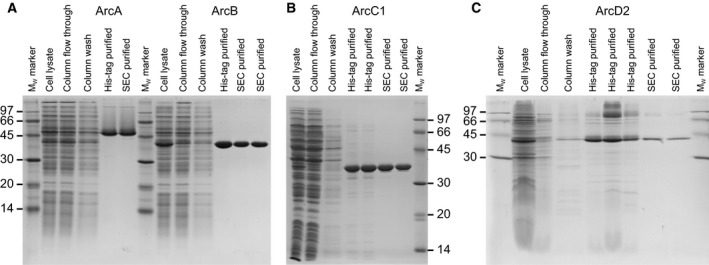
Full SDS/PAA gels showing the purification of ArcA, ArcB, ArcC1, and ArcD2. Molecular weight (*M*_W_) markers at 97, 66, 45, 30, 20, and 14 kDa. Cell lysate fractions equals raw cell lysate before purification, column flow through, and column wash fractions of the Ni^2+^–Sepharose resin purifications. Metal‐affinity and SEC purified fractions are also shown. (A) SDS/PAA gel for ArcA and ArcB. (B) SDS/PAA gel for ArcC1. (C) SDS/PAA gel for ArcD2.

Protein purity was determined with SDS/PAGE, using a 4% stacking gel and 12.5% separating gel, as described in Ref. [56]. ArcA, ArcB, and ArcC1 were purified in 50 mm KPi, pH 7.0, with 200 mm NaCl [plus 10% (v/v) glycerol for ArcC1] at concentrations between 4 and 8 mg·mL^−1^ and stored in 50 mm KPi, pH 7.0, with 100 mm NaCl [plus 10% (v/v) glycerol for ArcC1] at −80 °C. ArcD2 was purified in 50 mm KPi, pH 7.0, with 200 mm KCl plus 0.02% (w/v) *n*‐dodecyl‐β‐d‐maltoside and reconstituted into preformed liposomes (for lipid composition see below, Table 2). ArcD2 proteoliposomes were stored in 50 mm KPi, pH 7.0, in liquid nitrogen. Cell lysate at 10 mg·mL^−1^ and Ni^2+^–Sepharose resin flow through fractions were diluted 15‐fold, Ni^2+^–Sepharose resin wash fractions were diluted 1.25‐fold, and protein fractions were diluted to 0.2 mg·mL^−1^ with loading buffer [final concentrations: 2% (v/v) SDS, 4% glycerol, 0.01% bromophenol blue plus 5% β‐mercaptoethanol in 50 mm Tris/HCl, pH 6.8]. Protein gels were stained with Prosieve EX Safe Stain (Lonza Group Ltd, Basel, Switzerland) (Fig. 7). Protein concentration was calculated from the extinction coefficient and the absorbance at 280 nm, measured with a microvolume UV–Vis spectrophotometer (NanoDrop Technologies, Inc., Wilmington, DE, USA).

**Table 2:** Lipid compositions used in this study. All values are given in mole%.

Name	DOPE	DOPG	DOPC
50% DOPG	50	50	0
38% DOPG	50	38	12
25% DOPG	50	25	25
13% DOPG	50	13	37
0% DOPG	50	0	50
	POPE	POPG	POPC
38% POPG	50	38	12

### Oligomeric state of proteins

**Fig. 8:**
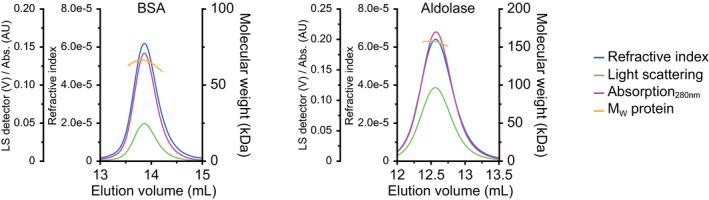
Static light scattering chromatograms of BSA and aldolase. Static light scattering (SLS) chromatograms for BSA (left) and aldolase (right), with refractive index (blue traces), light scattering (green traces), and absorption at 280 nm (pink traces). The molecular weight at the peaks (yellow traces) indicates 66 and 157 kDa for BSA and aldolase, respectively. BSA was used as a standard protein for ArcA, ArcB, and ArcC1; aldolase was used for ArcD2.

Ni^2+^–Sepharose/size‐exclusion chromatography‐purified fractions of ArcA, ArcB, ArcC1, and ArcD2 were analyzed on a second Superdex 200 Increase 10/300 GL size‐exclusion column (GE Healthcare, Chicago, IL, USA) in 50 mm KPi, pH 7.0, plus 100 mm NaCl [with 0.02% (w/v) DDM for ArcD2], which was coupled to a multi‐angle light scattering system with detectors for absorbance at 280 nm (Agilent Technologies, Inc., Santa Clara, CA, USA), static light scattering (Wyatt Technology Corporation), and differential refractive index (Wyatt Technology Corporation, Santa Barbara, CA, USA). Data analysis was performed with the astra software package (Wyatt Technology Corporation), using a value for the refractive index increment (dn/dc)_protein_ of 0.180 mL·mg^−1^ and (dn/dc)_detergent_ of 0.143 mL·mg^−1^ [41]. We calibrated the detectors with BSA for ArcA, ArcB, and ArcC1 and with aldolase for ArcD2 (Fig. 8).

### Enzymatic assays for ArcA and ArcB

Activity of ArcA and ArcB was measured with the COLDER assay, originally described in Ref. [48]. The values for *K*_M_ and *V*_MAX_ data were obtained as described in Ref. [38]. Briefly, either 2 µg·mL^−1^ ArcA or 0.25 µg·mL^−1^ ArcB was incubated in 50 mm KPi, pH 7.0, at 30 °C for 3 min in a total volume of 275 µL. To start the reaction, varying concentrations of either 0–480 µm l‐arginine for ArcA, or 0–10 mm l‐ornithine plus 0–5 mm carbamoyl‐Pi for ArcB were added and citrulline formation was analyzed every 30 s over a 2.5‐min period. About 200 µL of COLDER solution [20 mm 2,3‐butanedione monoxime (DAMO), 0.5 mm thiosemicarbazide, 2.25 m phosphoric acid, and 4.5 m sulfuric acid plus 1.5 mm ammonium iron(III) sulfate] was pipetted into each well of a 96‐well flat‐bottom transparent polystyrene plate (Greiner Bio‐One International GmbH, Kremsmünster, Austria), to which 50 µL of reaction mixture was added to stop the enzymatic conversion. Additionally, a set of calibration samples with l‐citrulline concentrations ranging from 0 to 250 µm was added to the COLDER solution. To allow color development, the plate was sealed with thermoresistant tape (Nalge Nunc International, Rochester, NY, USA) and incubated at 80 °C for 20 min in a block heater (Cole‐Parmer Instrument Co Ltd, Saint Neots, UK). Afterward, the plate was cooled down to room temperature for 30 min, the condensate was centrifuged (1 min, 1000 ***g***, 20 °C), and the absorbance was measured at 540 nm in a plate reader (BioTek Instruments, Inc., Winooski, VT, USA). Enzyme activity (in nmol l‐citrulline·min^−1^·mg protein^−1^) was determined by the formula:

(1) $${\text{Act}}_{\text{enz}}=\frac{\Delta \text{enz}}{\Delta \text{cal}}\times \frac{1}{c_{\text{enz}}\times {\text{vol}}_{\text{rm}}},$$

where Δenz (AU·min^−1^) and Δcal (AU·nmol l‐citrulline^−1^) are the slopes of the enzyme and calibration curves, respectively; *c*_enz_ is the final concentration of enzyme in mg·mL^−1^ and vol_rm_ is the volume of the reaction mixture in mL.

After plotting the enzyme activities (reaction rates) at various concentrations of substrate, the datapoints were fitted with the Michaelis–Menten equation:

(2) $$v=\frac{V_{\text{MAX}}\times \left[S\right]}{K_{M}+\left[S\right]},$$

where *v* is the reaction rate, *V*_MAX_ is the maximum reaction rate, *K*_M_ is the Michaelis constant, and [*S*] is the substrate concentration. In addition, we used the Michaelis–Menten equation with uncompetitive inhibition to fit the substrate inhibition of ArcA, for which we modified Eqn (2) as follows:

(3) $$v=\frac{V_{\text{MAX}}\times \left[S\right]}{K_{M}+\alpha \times \left[S\right]},$$

where α is defined as:

(4) $$\alpha =1+\frac{\left[I\right]}{K_{I}},$$

where [*I*] is the apparent inhibitor concentration and *K*_I_ is the inhibition constant.

To determine the pH and temperature dependence, as well as the role of small molecule effectors, experiments were done following the same protocol, but with either 480 µm l‐arginine for ArcA or 5 mm l‐ornithine plus 5 mm carbamoyl‐Pi for ArcB as the substrates and with the following adjustments. For the experiments with ADI pathway intermediates, 0–10 mm of l‐ornithine, carbamoyl‐Pi, or l‐arginine was added to the reaction mixtures. To determine the pH dependence of the enzymes, the 50 mm KPi buffer in the reaction mixture was adjusted to a pH between 5.8 and 8.0 by mixing the appropriate amounts of KH_2_PO_4_ and K_2_HPO_4_. To determine the temperature dependence of the enzymes, the reaction mixture was incubated between 26 °C and 62 °C. To determine the effect of freeze–thaw cycles on the enzymes, the protein stocks (4.2–7.2 mg of protein·mL^−1^) were either not frozen (control sample) or frozen in liquid nitrogen and thawed in an ice‐water bath at 10 °C; the freeze–thaw cycles were repeated up to 8 times prior to determining the enzymatic activity. Alternatively, 2 µm ArcB was encapsulated into ArcD2 proteoliposomes (66 µL, 6.6 mg of lipid) with 5 mm Mg‐ADP (MgSO_4_ plus ADP) and 0.5 mm l‐ornithine in 50 mm KPi, pH 7.0, and the samples were frozen and thawed 0–5 times. Next, the proteoliposomes were dissolved with 0.1% (v/v) Triton X‐100 and the enzymatic activities of ArcA and ArcB were determined; control experiments showed that Triton X‐100 had no effect on the enzymes or the COLDER assay. To determine the salt dependence of the enzymes, 50 mm KPi, pH 7.0, was supplemented with 50–300 mm of KCl or NaCl. Alternatively, the reaction mixture was composed of 50–300 mm of KPi, NaPi, or Tris/HCl at pH 7.0.

### Enzymatic assays for ArcC1

The activity of ArcC1 was obtained from changes in ATP/ADP ratio as measured with the ratiometric fluorescent protein PercevalHR [50], as described in Ref. [38], or by direct measurements of ATP with the ATPlite™ Luminescence Assay System (PerkinElmer, Inc., Waltham, MA, USA). For the ATP luminescence measurements, 0.25 µg·mL^−1^ ArcC1 was incubated in 50 mm KPi, pH 7.0 (buffer A), at 30 °C for 3 min, in a total volume of 275 µL. To start the reaction, 5 mm Mg‐ADP plus 5 mm carbamoyl‐Pi were added and ATP formation was analyzed every 30 s for up to 2.5 min. To stop the reaction, 50 µL of the mixture was pipetted into 50 µL of ATPlite™ mammalian cell lysis solution plus 50 µL of buffer A, which was present in each well of a 96‐well flat‐bottom white polystyrene plate (Greiner Bio‐One International GmbH). Additionally, a set of calibration samples with Mg‐ATP concentrations ranging from 0 to 0.5 mm was added to the ATPlite™ mammalian cell lysis solution. The plate was then shaken for 5 min on an orbital shaker at 700 r.p.m., after which 50 µL of ATPlite™ substrate solution was added and the plate was shaken for another 5 min at 700 r.p.m. Next, the plate was dark adapted for 10 min before luminescence was read in a plate reader (BioTek Instruments, Inc.). Enzyme activity (in nmol Mg‐ATP·min^−1^·mg protein^−1^) was determined by Eqn (1) with the modification that Δcal is the slope of the calibration curve in AU·nmol Mg‐ATP^−1^.

For the experiments with ADI pathway intermediates, 0–10 mm of l‐arginine, l‐ornithine, or l‐citrulline was added to the reaction mixtures. To determine the effects of pH, temperature, freeze–thaw cycles, salt concentration, and buffer composition, the same adjustments were made as described in the previous section for ArcA and ArcB.

### Transport assays for ArcD2

The activity of ArcD2 was measured from the uptake of radiolabeled substrates, as described in Ref. [38], with some adjustments. Proteoliposomes with ArcD2 in the membrane at a protein‐to‐lipid ratio of 1 : 200 (w/w) (66 µL, 6.6 mg of lipid) were encapsulated with l‐ornithine (0, 0.5, 1, 2, 3.5, 5, or 10 mm) or l‐citrulline (0, 1, 2, 5, 10, or 20 mm) in buffer A with 5 freeze–thaw cycles in a total volume of 200 µL. The proteoliposomes were extruded 13 times through a 200‐nm pore size polycarbonate filter and diluted to 6 mL in buffer A with or without the same concentration of l‐ornithine or l‐citrulline as present on the inside. Proteoliposomes were collected by centrifugation (20 min, 225 000 ***g***, 4 °C), washed with buffer A (6 mL), centrifuged again, and resuspended in 30 µL buffer A per 6.6 mg of lipid, yielding a final concentration of 220 mg of lipid·mL^−1^. For the transport assay, proteoliposomes were diluted 100‐fold to a final concentration of 2.2 mg of lipid·mL^−1^ in pre‐heated (30 °C, 3 min) buffer A with 25 µm l‐arginine [of which 4% (mol·mol^−1^) was ^14^C‐radiolabeled] and 100 µL of samples was taken every 20 s over an 80‐s period. After given reaction times, the samples were diluted into 2 mL of ice‐cold buffer A and filtered over 0.45‐µm pore size cellulose nitrate filters to stop the uptake of amino acid. The filter was then washed with another 2 mL of buffer A. Radioactivity on the filter was quantified by liquid scintillation counting using Ultima Gold MV scintillation fluid (PerkinElmer) and a Tri‐Carb 2800TR scintillation counter (PerkinElmer). To impose a membrane potential, proteoliposomes were diluted 100‐fold into 50 mm NaPi, pH 7.0 (ΔΨ = −120 mV) instead of buffer A, supplemented with 1 µm of the potassium ionophore valinomycin.

To determine the *K*_M_ and *V*_MAX_ values of arginine/ornithine and arginine/citrulline transport, the same protocol was used except that the external concentration of arginine or the internal concentration of ornithine or citrulline were varied. To determine the optimal reconstitution conditions, ArcD2‐to‐lipid ratios of 1 : 800 (w/w), 1 : 400, 1 : 200, and 1 : 100 were used, and the proteoliposomes were filled with 10 mm l‐ornithine. To determine the lipid dependence of ArcD2, the protein was reconstituted into the lipid mixtures specified in Table 2; the proteoliposomes were filled with 10 mm l‐ornithine and were assayed with 10 µm l‐arginine on the outside [of which 10% (mol·mol^−1^) was ^14^C‐radiolabeled].

To determine the influx of arginine (without counter‐substrate present on the inside), the proteoliposomes were filled with buffer A and frozen‐thawed as described heretofore. Proteoliposomes were incubated in buffer A or 50 mm NaPi, pH 7.0, plus 1 µm valinomycin and were assayed with 1 µm ^14^C‐l‐arginine. Samples were taken every 10–15 min; the transport reactions were terminated and the vesicles were collected by ultrafiltration as described above. To determine the duration of the artificially imposed membrane potential, proteoliposomes were filled with 10 mm l‐citrulline and diluted in 50 mm NaPi, pH 7.0, with 1 µm valinomycin with or without 250 mm NaCl. 25 µm ^14^C‐l‐arginine was added 0, 10, 20, 30, 45, and 60 min after the imposition of the membrane potential.

## Conflict of interest

The authors declare no conflict of interest.

## Author contributions

BP, TP, and SS designed the research; BFG, CD‐D, and TP performed most of the research; SS purified enzymes; BFG, BP, CD‐D, SS, and TP analyzed data; and BP, SS, and TP wrote the paper.
