# Supplementary material for: Enzymology of the pathway for ATP production by arginine breakdown
Source: FEBS J. 2020 May 5;288(1):293–309. doi: 10.1111/febs.15337 (PMC7818446; doi:10.1111/febs.15337)
Supplement: Supplementary file 1 [file FEBS-288-293-s001.zip › febs15337-sup-0001-FigS1-S2.pdf]

Supplementary information

Enzymology of the pathway for ATP production by arginine breakdown

Tjeerd Pols, Shubham Singh, Cecile Deelman-Driessen, Bauke F. Gaastra and Bert Poolman

Department of Biochemistry, Groningen Biomolecular Sciences and Biotechnology Institute & Zernike Institute for Advanced Materials, University of Groningen, Nijenborgh 4, 9747 AG Groningen, The Netherlands.

*Correspondence to: b.poolman@rug.nl

Supplementary Figures 1-2

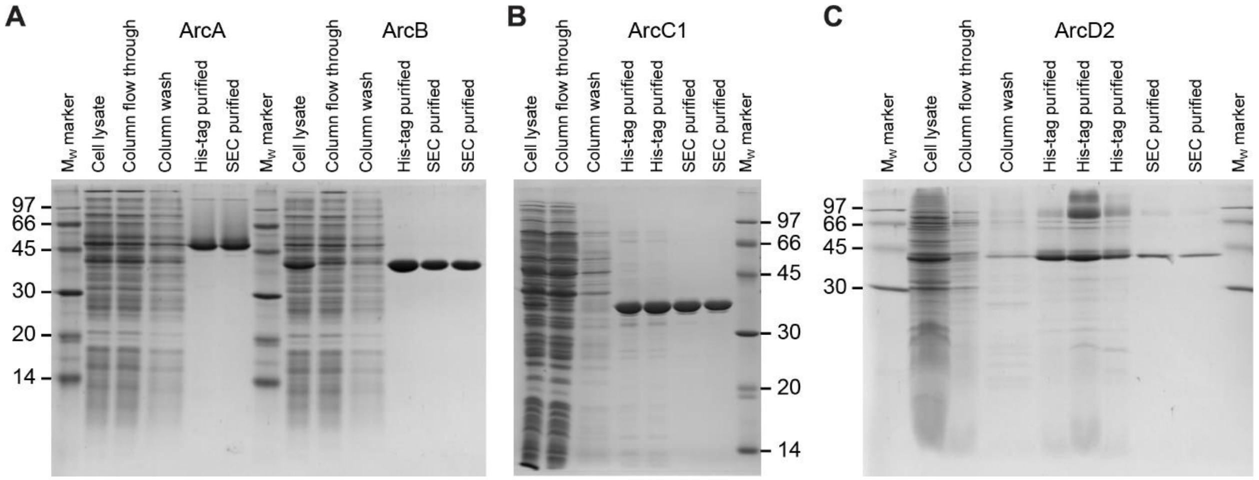

Supplemental Figure S1. Full SDS-PAA gels showing the purification of ArcA, ArcB, ArcC1 and ArcD2. Molecular weight (M_W) markers at 97, 66, 45, 30, 20 and 14 kDa. Cell lysate fractions equals raw cell lysate before purification, column flow through, and column wash fractions of the Ni^{2+}-Sepharose resin purifications. Metal-affinity and size-exclusion chromatography (SEC) purified fractions are also shown. (**A**) SDS-PAA gel for ArcA and ArcB. (**B**) SDS-PAA gel for ArcC1. (**C**) SDS-PAA gel for ArcD2.

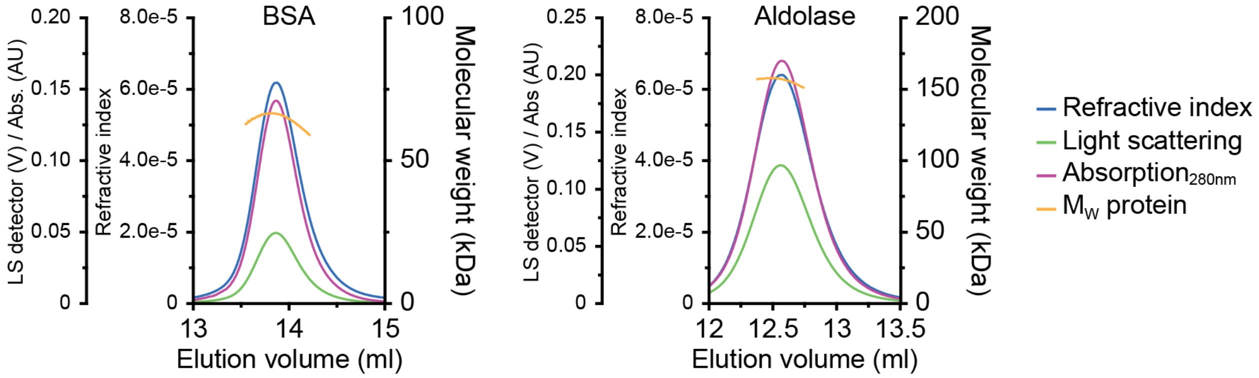

Supplemental Figure S2. Static light scattering chromatograms of BSA and aldolase. Static light scattering (SLS) chromatograms for bovine serum albumin (BSA; left) and aldolase (right), with refractive index (blue traces), light scattering (green traces) and absorption at 280 nm (pink traces). The molecular weight at the peaks (yellow traces) indicate 66 and 157 kDa for BSA and aldolase, respectively. BSA was used as a standard protein for ArcA, ArcB and ArcC1; aldolase was used for ArcD2.
